# The Therapeutic Potentials of Ayahuasca: Possible Effects against Various Diseases of Civilization

**DOI:** 10.3389/fphar.2016.00035

**Published:** 2016-03-02

**Authors:** Ede Frecska, Petra Bokor, Michael Winkelman

**Affiliations:** ^1^Department of Psychiatry, Faculty of Medicine, University of DebrecenDebrecen, Hungary; ^2^Doctoral School of Psychology, University of PécsPécs, Hungary; ^3^School of Human Evolution and Social Change, Arizona State University, TempeAZ, USA

**Keywords:** addiction medicine, ayahuasca, diseases of civilization, dimethyltryptamine, oxidative stress

## Abstract

Ayahuasca is an Amazonian psychoactive brew of two main components. Its active agents are β-carboline and tryptamine derivatives. As a sacrament, ayahuasca is still a central element of many healing ceremonies in the Amazon Basin and its ritual consumption has become common among the mestizo populations of South America. Ayahuasca use amongst the indigenous people of the Amazon is a form of traditional medicine and cultural psychiatry. During the last two decades, the substance has become increasingly known among both scientists and laymen, and currently its use is spreading all over in the Western world. In the present paper we describe the chief characteristics of ayahuasca, discuss important questions raised about its use, and provide an overview of the scientific research supporting its potential therapeutic benefits. A growing number of studies indicate that the psychotherapeutic potential of ayahuasca is based mostly on the strong serotonergic effects, whereas the sigma-1 receptor (Sig-1R) agonist effect of its active ingredient dimethyltryptamine raises the possibility that the ethnomedical observations on the diversity of treated conditions can be scientifically verified. Moreover, in the right therapeutic or ritual setting with proper preparation and mindset of the user, followed by subsequent integration of the experience, ayahuasca has proven effective in the treatment of substance dependence. This article has two important take-home messages: (1) the therapeutic effects of ayahuasca are best understood from a bio-psycho-socio-spiritual model, and (2) on the biological level ayahuasca may act against chronic low grade inflammation and oxidative stress via the Sig-1R which can explain its widespread therapeutic indications.

## Introduction

Ayahuasca, a psychoactive Amazonian sacrament, has raised increased scientific and lay interest during the last two decades. Traditionally ayahuasca has been used in Ecuador, Columbia, Peru, and Brazil, where it is also known as *natema, hoasca, daime, yagé*, or *yajé* The decoction is prepared by simultaneously boiling two admixture plants, the *Banisteriopsis caapi (Malpighiaceae)* containing β-carboline type alkaloids such as harmine and tetrahydroharmine; and most commonly *Psychotria viridis (Rubiaceae)*, which provides the psychoactive alkaloid DMT (McKenna, 2004; Szára, 2007). Sometimes *Psychotria viridis* is substituted by other DMT containing plants such as *Diplopterys cabrerana (formerly B. rusbyana)* of the family *Malpighiaceae*. The name ayahuasca is a compound word in Quechua language, where *aya* means soul, ancestors or dead persons and *wasca* (*huasca*) means vine or rope (Luna, 2011). Therefore, the most prevalent translation of the word is “vine of the soul”. Skeptics may prefer the other linguistic alternative: “rope of death”, but this paper will provide arguments favoring the former interpretation above the latter one.

Ayahuasca has been used as a central element of religious, magical, curative, initiation, and other tribal rituals for millennia (Naranjo, 1986), originally by the indigenous groups and later by the mestizo populations of the region, who respect the brew as a sacrament and value it as a powerful medicine. The indigenous and mestizo communities regularly use ayahuasca to treat physical ailments, mental problems and frequently handle their social issues, spiritual crises with the help of the brew. A Peruvian tradition called *vegetalismo* regards ayahuasca as one of the teacher plants that convey knowledge to humans (Luna, 1986), and considers the experience induced by its ingestion *trabajo* (work). In addition to its traditional and mestizo uses, ayahuasca also forms a central component of the rituals of three Brazilian syncretic churches: the *Santo Daime*, the *União do Vegetal* and the *Barquinha*. The history of these churches dates back to the first half of the 20th century, and by now they are present in 23 countries (de Rios and Rumrrill, 2008; Liester and Prickett, 2012). Obviously there is a striking discrepancy between the indigenous South American and official Western view^1^ on ayahuasca use, which calls for scientific explanation and a healthy resolution.

Due to the growing popularity of the sacrament, masses of people from all parts of the world travel to the Amazon to participate in ayahuasca rituals. This unique phenomenon characterized by some as “drug tourism” (de Rios, 1994) is not as frivolous pursuit as it sounds (Grunwell, 1998), since a significant number of travelers searches for spiritual and therapeutic opportunities. The principal motivations can be characterized as: seeking improved insight, personal growth; emotional healing; and contact with a sacred nature, deities, spirits and natural energies produced by the ayahuasca (Winkelman, 2005). The trend of popularization—known as the “globalization of ayahuasca”—flows both ways, as this Amazonian tradition spreads beyond its native habitat and gets adopted into non-indigenous circles of the Western world (Tupper, 2008) either within or outside of the context of syncretic churches.

During the last couple of years several publications have been written with the goal to summarize our knowledge about ayahuasca from various perspectives (see in Labate and Cavnar, 2014). The primary aim of this article is to give an overview about the facts and hypotheses related to the possible therapeutic mechanisms of the brew in light of recent advances of the field; with the secondary aim of addressing its known adverse effects. By adhering to a biopsychosociospiritual model (Bishop, 2009) the authors will explore every level in the following order: starting with biochemistry, neuropharmacology, physiology, brain imaging, then moving to the psychological effects, social ramifications, and finally addressing spiritual implications. Efforts are taken to keep a balance among the biomedical, psycho-social and spiritual aspects of healing since “*Madre Ayahuasca, la sagrada medicina* (Mother Ayahuasca, the sacred medicine)” is best understood within this quadrilateral framework.

## The Neurobiological Background of Ayahuasca

From a pharmacological perspective the main ingredients of ayahuasca are DMT and the β-carboline derivative alkaloid harmine, harmaline, and tetrahydroharmine (Callaway et al., 1996). The harmine, tetrahydroharmine, and harmaline work as reversible inhibitors of the A-type isoenzyme of the monoamine oxidase (MAO), while tetrahydroharmine also exerts selective serotonin reuptake inhibitor (SSRI) effects (dos Santos, 2010). The hallucinogenic component DMT is abundant in the plant kingdom (Khan et al., 2012) and it is also present in mammalian organism; studies have detected it in human blood, brain, cerebrospinal fluid (Wallach, 2009), and the pineal gland of rats (Barker et al., 2013). While DMT is classified as an endogenous hallucinogen, together with bufotenin and 5-methoxy-DMT (Christian et al., 1977; Hollister, 1977), its exact function is yet unclear (Barker et al., 2012).

More than 50 years of research has proven to be insufficient to provide a proper neurobiological description of the role of endogenous hallucinogens. This is in part due to a paradigm problem in which these natural substances with many biological functions have been primarily studied in terms of being “hallucinogens,” producing false perceptions. It is obvious that these substances play a role in producing alterations of consciousness such as dreaming, psychosis, and near death experience (Strassman, 2001). These effects presumably reflect action on serotonin (5-HT) receptors (5-HT1A, -2A and -2C) as well as the trace amine associated receptors (supposedly TAAR6) (Wallach, 2009). While the scientific knowledge about trace amine associated receptors is rapidly increasing, it is still deficient. However, the Sig-1R action of DMT may turn out to be more revealing about its physiological function (see below).

**FIGURE 1:**
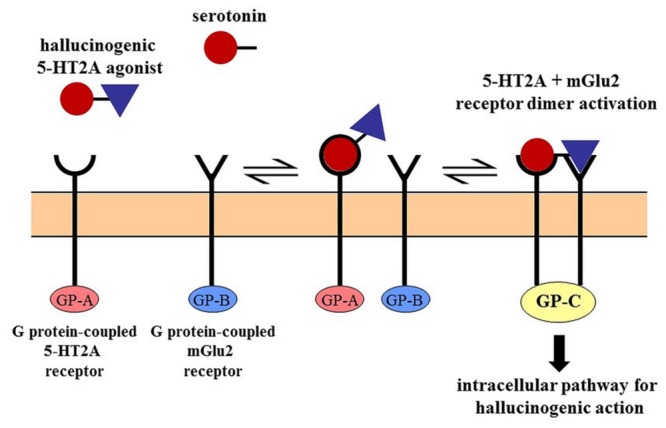
**Cross-talk between receptors by dimerization.** The 5-HT2A receptor mediated hallucinogen-specific intracellular pathway requires the dimerization of the 5-HT2A receptor with the mGlu2 receptor. This unique (G protein-coupled) pathway is associated only with the dimer and not activated by either receptor alone. Serotonin lacks the mGlu2 receptor binding feature and the psychotropic effects of hallucinogens are abolished by the elimination of the mGlu2 receptor. The 5-HT2A-mGlu2 dimer is the prime target of some serotonergic hallucinogens (Moreno et al., 2011).

Dimethyltryptamine exerts anxiolytic effects through 5-HT1A receptor agonism (Jacob and Presti, 2005), and its psychedelic effect is connected to its 5-HT2A receptor-activating capacity (Nichols, 2004). However, simple 5-HT receptor mediated actions are not sufficient to explain drug-induced hallucinations since 5-HT itself, and some 5-HT2A agonists (i.e., lisuride) are not hallucinogenic. Over the past two decades, it became clear that different agonists having similar binding affinities for the same sites, could elicit distinct signaling pathways within the cell. These observations are interpreted on the basis of receptor–receptor and ligand–receptor interactions such as “receptor oligomerization,” “receptor trafficking,” or “biased agonism” (Moreno et al., 2011; Borroto-Escuela et al., 2014) which activate different G proteins resulting in divergent intracellular cascades. **Figure 1** schematically illustrates the mechanism of receptor dimerization wherein metabotropic glutamate (mGlu2) receptors belonging to an entirely separate receptor family form a complex with the 5-HT receptor and trigger an intracellular pathway for hallucinogenic action. This may explain why lisuride which has a similar receptor binding profile to the chemically similar ergoloid lysergic acid diethylamide (LSD), lacks the psychedelic effects of its sister compound (Rogawski and Aghajanian, 1979). In case of DMT, a recent study (Carbonaro et al., 2015) concluded that while 5-HT2A receptors play a major role in mediating its effects, mGluR2 receptors likely modulate the action. Unlike the related tryptamine derivative psilocybin, DMT does not precipitate tolerance upon repeated use (Strassman et al., 1996); this produces further complications for simple receptor-based interpretations.

The latest identified target for DMT’s action is the Sig-1R. Sigma receptors were originally misclassified as opioid receptors but later turned out to be non-opioid receptors of their own type. The Sig-1R subclass has been demonstrated to consist of chaperone molecules concentrated in normal cells of the brain, retina, liver, lung, heart, immune system, but also in many tumor lines (Maurice and Su, 2009). Chaperones are proteins that assist the correct folding of other protein clients. The Sig-1R chaperon has many unique features with an amino acid sequence distant from mammalian proteins and homologous to fungal sterol isomerases (Moebius et al., 1997). Sig-1R sites are concentrated in the human brain with the highest densities in the cerebellum, nucleus accumbens, and cerebral cortex (Weissman et al., 1988). Inside the cell Sig-1R is located mainly at the ER–mitochondrion interface—referred to as the MAM—and regulates cellular bioenergetics, particularly under stressful conditions (Su et al., 2010; Mori et al., 2013; Hayashi, 2015). There is another mode of Sig-1R action at the plasma membrane where it translocates under stimulation by agonists.

As an intracellular receptor localized at the MAM, Sig-1R integrates many signaling pathways and serves as a “tunnel” for lipid transport and Ca^2+^-signaling between the ER and mitochondria (Hayashi and Su, 2007). Its involvement is critically in ion channel activities and neuronal differentiation. The wide scope and effect of ligand binding to Sig-1R indicate that Sig-1Rs are intracellular signal transduction amplifiers (Su and Hayashi, 2003). The ER-mitochondrion interface at the MAM serves as an important subcellular entity in the regulation of cellular survival via Sig-1R by enhancing the stress–response signaling (Mori et al., 2013). Sig-1R also protects the cells against reactive oxygen species and activates the antioxidant response elements (Pal et al., 2012), therefore Sig-1R agonists such as DMT may function as indirect antioxidants. More interestingly the induction of Sig-1R can repress cell death signaling: up-regulation of Sig-1R suppresses the apoptosis caused by ER stress (Omi et al., 2014). Tryptaminergic trace amines as well as neurosteroids (e.g., dehydroepiandrosterone, pregnenolon) are endogenous ligands that activate the Sig-1R (Fontanilla et al., 2009).

The Sig-1R ER chaperone function is essential for the metabotropic receptor signaling and for the survival against cellular, particularly ER stress. Dysfunctional chaperones are responsible for numerous diseases (Tsai et al., 2009). Altogether, no other receptor has ever been associated with so many different diseases as the Sig-1R. It has so far been implicated in illnesses like Alzheimer’s disease, Parkinson’s disease, cancer, cardiomyopathy, retinal dysfunction, perinatal and traumatic brain injury, frontal motor neuron degeneration, amyotrophic lateral sclerosis, HIV-related dementia, major depression, and psychostimulant addiction (Su, 2015). How those two modes of actions of the Sig-1R may relate to this plethora of diseases remain to be clarified but its protective influence has been verified on various aspects of cellular processes, such as calcium signaling, mitochondrial functions, ER stress, survival and apoptotic pathways (to be discussed later), and tumor cell proliferation (Tsai et al., 2014). As Sig-1Rs are expressed in the immune system, immunomodulatory functions have also been reported in the literature (Szabo et al., 2014; Dong et al., 2015).

## The Possible Role of DMT in Tissue Protection and Neuroregeneration

Dimethyltryptamine is considered as a natural ligand, an endogenous agonist of the Sig-1R (Fontanilla et al., 2009). It is assumed that the Sig-1R might be involved in the DMT-induced psychedelic effects (Su et al., 2009); however, this is somewhat counterintuitive since many drugs—including non-hallucinogens—bind promiscuously to the Sig-1R with higher affinity than DMT. The results of a recent surge in Sig-1R research are pointing toward a different horizon by outlining a physiological role of DMT instead of the long-held pathological view. If the Sig-1R promotes cellular (neuronal) survival against oxidative stress (Pal et al., 2012) and regulates immune processes (Jarrott and Williams, 2015), one may attribute the same physiological role to its endogenous ligands, like DMT (Frecska et al., 2013). Since the Sig-1R is also known to regulate morphogenesis of neuronal cells, such as neurite outgrowth, synaptogenesis, and myelination (Ruscher and Wieloch, 2015); neurorestorative effects are reasonably expected from DMT. In a previous paper (Frecska et al., 2013) we concluded that the function of DMT may extend central nervous activity and involve a more universal role in cellular protective mechanisms. We provided converging evidence that while DMT is a substance which produces powerful psychedelic experiences, it is better understood not as a hallucinogenic drug of abuse, but rather an agent of significant adaptive mechanisms like neuroprotection, neuroregeneration, and immunity.

Nevertheless, immunoregulation by DMT is a bidirectional process. Sig-1Rs are expressed together with 5-HT receptors in immune cells conveying both inflammatory and anti-inflammatory signals (Szabo, 2015). These receptors are essential in the “fine-tuning” of innate and adaptive immune responses. Human clinical studies showed that ayahuasca can alter the number and distribution of blood immune cells in a way that can increase the antiviral and anti-tumor immunity of the consumer (reviewed in Frecska et al., 2013). Ayahuasca also influences the distribution of lymphocyte subpopulation: CD4 lymphocytes decrease and the number of natural killer cells increase significantly with time (dos Santos et al., 2012). The possible anti-cancer activity of the decoction makes it a promising candidate for further researches in novel pharmacotherapies (Schenberg, 2013). Furthermore, DMT may also be an adaptogen increasing the survival rate of neurons or other cell types during acute hypoxia or under chronic oxidative stress.

## Mechanisms Proposed as a Basis for Ayahuasca’s Effects on Systemic and Degenerative Illnesses

Chronic LGI is becoming widely accepted as a common basis for many diseases of civilization (Ruiz-Núñez et al., 2013; **Table 1**). Repeated psychological stress, constant environmental pollution, and smoking behavior are associated with chronic LGI, which is one of the main causes of insulin resistance that is the pathological foundation of metabolic diseases (Aseervatham et al., 2013). Moreover, chronic LGI is involved in all stages of the atherosclerotic process and is being increasingly recognized as a universal mechanism in various chronic degenerative diseases, such as autoimmune diseases, certain cancers, neuropsychiatric diseases (e.g., Alzheimer’s disease, Parkinson’s disease, major depression), and osteoporosis. Dysbiosis of the gut microbiota with increased intestinal permeability (“leaky gut”) are all possible root causes of LGI (Carrera-Bastos et al., 2011).^2^

**Table 1:** Common diseases of civilization.

• Metabolic syndrome	• Neurodegenerative diseases
• Cardiovascular diseases	• Alzheimer’s dementia
• Cancer	• Autism
• Osteoarthrosis, osteoporosis	• Depression
• Autoimmune disorders	• Schizophrenia
• Chronic obstructive pulmonary disease	• Attention deficit hyperactivity disorder
• Macular degeneration	• Chronic pain syndrome

*The table lists those illnesses which—due to different diet, lifestyle, and environmental factors—are more prevalent in advanced agricultural and industrial societies as compared to hunter-gatherers, traditional pastoralists and horticultural communities, or have poorer outcome in the urban environment (after; Carrera-Bastos et al., 2011; Aseervatham et al., 2013; Ruiz-Núñez et al., 2013; Srivastava and Kumar, 2015).*

Chronic LGI is closely related to oxidative stress and ER stress, and together they form a molecular web, a network interwoven with loops exacerbating each other (Chaudhari et al., 2014). Regulation of protein folding homeostasis (proteostasis) is essential for the execution of fundamental cellular functions. ER is the cellular organelle responsible for this role. Disturbance in protein folding is central to a large diversity of illnesses and growing evidences suggest ER stress as being a cardinal component in the development of a pathological condition (Díaz-Villanueva et al., 2015; Srivastava and Kumar, 2015). The cause of diseases may be various, but ER stress resulting from chronic LGI or oxidative stress may contribute to the severity and the poor prognosis of the diseased state. ER function can be altered and made dysfunctional by hypoxia, hyperglycemia, hyperlipidemia, viral infections, disturbances in cellular calcium levels, redox regulation, or by endogenous reactive oxygen species production (Chaudhari et al., 2014). These so-called stress signals exhaust the ER machinery and result in accumulation of unfolded proteins. An adaptive process called UPR is triggered with an aim to restore ER homeostasis. However, if the stress signal is severe and/or prolonged cell death pathways are elicited in form of apoptotic and pro-inflammatory reactions (Hiramatsu et al., 2015).

ER stress with UPR is thought to play a key role in neuropsychiatric illnesses such as Alzheimer’s disease, Parkinson’s disease, amyotrophic lateral sclerosis, bipolar disorder, and in other illnesses of civilization such as atherosclerosis, diabetes, cancer, autoimmune, and cardiovascular disorders (Chaudhari et al., 2014). All of these disorders may have common mechanism: failure of protein homeostasis. Deficits in ER-proteostasis lead to the formation of misfolded proteins characteristic of neurodegenerative diseases (Hetz and Mollereau, 2014; Plácido et al., 2014). Originally UPR has a cell protective effect: it prevents overload of ER lumen with newly synthesized proteins and activates degradation of misfolded proteins. However, misfolded proteins directly enter from ER into mitochondria and after prolonged UPR activation they cause dysfunction in energy production (Schon and Area-Gomez, 2013; Volgyi et al., 2015). Targeting Sig-1R by agonists may regulate ER stress and UPR, manage ER perturbations, regulate the formation of toxic misfolded proteins, and prevent the cell-killing apoptotic pathways (Rivas et al., 2015). Similar effects are expected from the endogenous Sig-1R ligand DMT.

**FIGURE 2:**
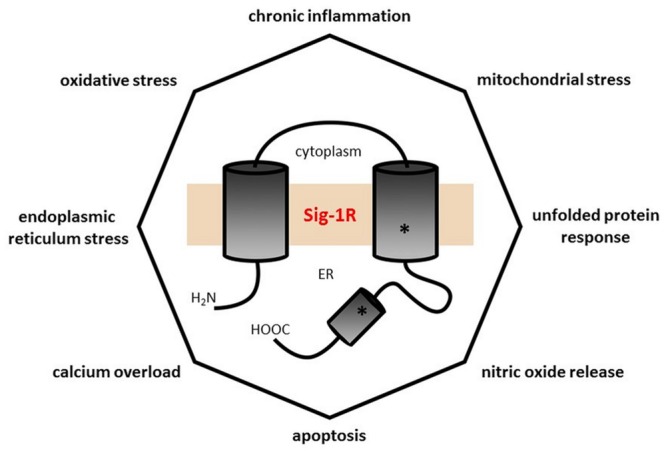
**The polygon of self-destructive forces.** Every angle of this octagon represents a pathophysiological process closely related to almost all of the others, and each pathological process is known to be involved in several illnesses of civilization (see **Table 1**) with extensive overlap (e.g., Alzheimer’s disease has chronic low grade inflammation, increased nitric oxide signaling, calcium dyshomeostasis, apoptosis, mitochondrial dysfunction, oxidative, and endoplasmic reticulum stress in its etiopathology). The central position of the Sig-1R illustrates its significant influence in mitigating these pathological processes. The number of angles is rather arbitrary: one may add others like insulin resistance, glutamate release, plasma membrane deficiency, etc.

Tracing Alzheimer’s disease, Parkinson’s disease, or major depression to inflammatory processes (chronic LGI), “leaky gut”, ER stress, protein folding deficit, glutamate excitotoxicity, mitochondrial dysfunction, calcium overload, death receptor pathways, and Sig-1R involvement means following the loops of the same web of interactions since all are different aspects of the same core phenomenon. The Sig-1R located at the MAM is an excellent candidate for interfering with the conversion of environmental stress in general and psychological stress in particular into cellular stress response by its regulatory effect on signaling between the ER and mitochondrion (Hayashi, 2015). This is the point where DMT and ayahuasca take their place in this puzzle via Sig-1R action. It seems to us that the ingenuity of South American people discovered a broad-spectrum remedy, which hits the dead center of the discussed vicious circle of the malfunctioning molecular web involved in oxidative stress (**Figure 2**).

There is more to ayahuasca’s therapeutic potentials besides its DMT content and above the neurobiological level. The psychological aspects will be discussed later. Here we address the other important active agents of ayahuasca, the β-carboline alkaloids, which act as selective, reversible MAO-A inhibitors (McKenna and Towers, 1984; dos Santos, 2010) with almost no effect on MAO-B (Herraiz et al., 2010). MAO inhibition is crucial as without the β-carbolines the DMT content of orally ingested ayahuasca would be broken down before crossing the blood-brain barrier. Moreover, the fact that MAO is located inside cells bound to the outer membrane of mitochondria in proximity of the Sig-1R raises the possibility that the synergy between the active compounds of ayahuasca happens not only at the periphery, but also inside neurons and glial cells. Without this intraneuronal MAO inhibition less DMT would reach the Sig-1R at the MAM. Furthermore, there are indications that the β-carboline alkaloids themselves have medicinal properties, such as anthelmintic (Hassan, 1967), antimicrobial (Ahmad et al., 1992), and vasorelaxant (Shi et al., 2001) effects, in addition to ethnopsychiatric (Shepard, 1998), sociopsychotherapeutic (Andritzky, 1989), and rehabilitative functions (Mabit et al., 1995). Harmala alkaloids have demonstrated strong psychoactive properties (Naranjo, 1967), and they act as stimulants on the central nervous system (Venault and Chapouthier, 2007). Osório’s team (Osório et al., 2011) attributed an observed antidepressant effect of ayahuasca (Osório et al., 2015) to these alkaloids, which is in line with ethnographic observations suggesting that many native users of ayahuasca ascribe sacramental respect to the *B. caapi* and not the DMT containing plant constituents.

From a biological standpoint the extent to which DMT and harmine play a role in ayahuasca effects is difficult to judge since the brew contains a significant amount of bioactive substances in addition to the indole and β-carboline alkaloids. An important example of such compounds is the group of antioxidant polyphenols, which can also be linked to the observed immunomodulatory effects (Szabo et al., 2014). Antioxidants are known for their capacity of reducing inflammatory processes or even stopping them (Grimble, 1994; Geronikaki and Gavalas, 2006). Malignant transformation is also inhibited by polynucleotides through providing protection against oxidative stress for other cellular compounds (Marquardt, 1984). In addition to the immunomodulatory effects, ayahuasca may also exhibit neuroprotective and neurorestorative qualities. Hence, it has been suggested that ayahuasca can be applied therapeutically in Parkinson’s and other neurodegenerative diseases (Samoylenko et al., 2010). Ayahuasca’s high content of bioactive materials points toward a combined mechanism of the various effect and calls for further clinical research to reveal the detailed pharmacology of the constituents.

## Vegetative and Adverse Effects of Ayahuasca

Serotonin stimulation is known to affect the whole organism not just the brain. It causes vegetative changes such as increase in systolic and diastolic blood pressure, pulse rate, provokes nausea, vomiting, and pupil dilation (Boyer and Shannon, 2005). Ayahuasca was found to significantly raise the systolic and diastolic blood pressure by 35 Hgmm and the pulse rate by 26 bpm in a 2 min time interval while the rate of increase declines upon repeated intake (Riba et al., 2001). Besides actions on the vegetative nervous system ayahuasca also induces endocrine response. Elevation in prolactin, cortisone, and growth hormone levels has been reported (dos Santos et al., 2011; dos Santos et al., 2012).

While the intravenously injected DMT can cause considerable cardiac stress, it is less burdensome for humans if taken orally. Based on animal research Gable (Gable, 2007) developed a model calculation that determined the median lethal dose of DMT in 8 mg/kg for oral ingestion in human subjects. The average ceremonial dose of DMT in ayahuasca preparations is about 27 mg; therefore, the safety margin for ayahuasca is approximately 20 (Gable, 2007). Scientific sources mention only one fatal case of ayahuasca consumption (Sklerov et al., 2005). The toxicological judgment based on uncontrolled street abuse is largely influenced by cases when extra ingredients other than the two basic components (e.g., datura or tobacco) are mixed into the decoction since the intoxicating effects of these extra ingredients can in turn be attributed to ayahuasca by the toxicological reports.

The MAO-A inhibition induced by the β-carboline alkaloids presumably results in an increased level of serotonin in the neural pathways, which theoretically can lead to serotonin syndrome in extreme cases (Callaway and Grob, 1998). However, the competitive, reversible nature of the inhibition may explain the lack of well documented serotonin syndrome cases despite the globalization of ayahuasca and probable inclusion of a large number of session participants taking SSRIs. What makes the issue more complicated is that at the time of onset, ayahuasca’s vegetative effects represent some sort of mild or moderate serotonin syndrome. Nevertheless, materials which may negatively interact with ayahuasca and capable to induce hyperserotonemia include ginseng (*Panax ginseng*), St John’s-wort (*Hypericum perforatum*), dextromethorphan, 3,4-methylenedioxy-*N*-methylamphetamine, SSRIs or MAO inhibitors (Callaway and Grob, 1998). Furthermore, cardiovascular or endocrine problems, abnormal lipid metabolism, glaucoma, fever, and pregnancy are contraindications for ayahuasca consumption (Gable, 2007).

A tendency for psychosis or family history of mental illness predispose ayahuasca for triggering a psychotic episode or long-term depersonalization syndrome. dos Santos and Strassman (2011) reported a case where a young male individual with prior experience in the use of psychedelic substances and having previous positive experience with ayahuasca fell repeatedly into psychosis after two *Santo Daim*e ceremonies. Even without proper screening the statistical probability of such cases seems to be low. The majority of the encountered problems originate from the unpreparedness of the participants, the inadequate setting (the parameters of the circumstances in which the ingestion of ayahuasca takes place), the lack of socio-cultural-cosmological embeddedness of the experiences or the lack of their integration afterward.

There is not yet any scientific evidence—or even personal reports—indicating that ayahuasca use elicits substance dependence. The ritual use of ayahuasca shows considerable differences to the traditional psychosocial harms of drug consumption (Fábregas et al., 2010). As with other psychedelic experiences an elevated emotional state may remain for a few days or weeks after ayahuasca consumption. In such cases life seems more beautiful and joyful, filled with a deeper meaning than before the experience. Therefore a feeling of emptiness or grayness can arise after the experience fades away but this is not generally the case.

## Central Nervous System Effects of Ayahuasca

Riba’s group (Riba et al., 2006) conducted single-photon emission computed tomography to reveal the brain areas affected by ayahuasca ingestion. Increased activity was recorded bilaterally in the anterior insula, in the fronto-medial cortical anterior cingulate of the right hemisphere and the left amygdala. The latter two play a role in the regulation of emotional arousal and the information processing of emotions. Moreover the anterior cingulate is involved in somatic attention and the experience of subjective feeling states (Bouso and Riba, 2011). By analyzing the binocular rivalry under the effect of the brew, conclusions were drawn that ayahuasca influences hemispheric dominance (Frecska et al., 2003) and gamma oscillations (Frecska et al., 2004).

Using functional magnetic resonance imaging technique de Araujo et al. (2012) found that ayahuasca induces a robust activation of occipital, temporal, and frontal areas during a closed-eyes imagery task. The consumption of the brew activated an extended network in the brain (that has previously been correlated to visual perception, memory and intention) in which the Brodmann areas BA10, BA17, BA30, and BA37 play a central role. The ayahuasca-produced stimulation of the primary visual cortex was comparable to the effect of natural images with the eyes open. Moreover, this effect correlated significantly with the occurrence of perceptual changes measured by rating scales. These authors concluded that by boosting the intensity of imagination to the same level of sensory perception ayahuasca lends a status of reality to inner experiences.

Several studies have employed electroencephalographic methods to record brain oscillatory activity after ayahuasca intake. Results indicated significant power increase in the slow gamma (36–44 Hz) band at left occipito-temporo-parietal electrodes, with tendencies to power decreases in theta and delta bands (Don et al., 1998). Another study revealed alpha power decrease at left temporal and centro-parietal sites peaking 90 min after ingestion, while decreases were found for delta and theta waves in the parietal areas (Riba et al., 2002). The analysis of Alonso’s group (Alonso et al., 2015) showed that ayahuasca preparation significantly changed the coupling of brain oscillations between anterior and posterior recording sites in the following pattern. Frontal structures decreased their influence over posterior (central, parietal, and occipital) sites which correlated with the intensity of subjective effects. On the other hand, posterior areas increased their influence over signals measured at anterior locations in parallel with the degree of incapacitation experienced (Alonso et al., 2015). These effects reflect the general action of psychedelics hypothesized by Winkelman (2007) in the proposed concept of psychointegrator, where the normal domination of cognition by frontal brain activity is replaced by intense discharges from the lower areas of the brain that are imposed on the frontal cortex.

Another recent project addressing the time course of ayahuasca effects on the electroencephalogram revealed a biphasic feature (Schenberg et al., 2015). After 50 min from ingestion of the brew there was observed a reduced alpha band activation over the left parieto-occipital cortex, followed by increased slow- and fast-gamma power (30–50 and 50–100 Hz, respectively) between 75 and 125 min. The slow-gamma power increase was located at the left centro-parieto-occipital, left fronto-temporal, and right frontal cortices while fast-gamma increases were significant at left centro-parieto-occipital, left fronto-temporal, right frontal, and right parieto-occipital areas. These effects correlated significantly with the circulating levels of ayahuasca’s main ingredients, such as DMT, harmine, tetrahydroharmine, and some of their metabolites. The significance of gamma power in the broader context of consciousness involves its role in binding of information across diverse regions of the brain and in driving theta wave responses.

Based on the conceptual framework of integrated information theory Gallimore (Gallimore, 2015) supposed that the promotion of gamma oscillations is responsible for the perceptual effects of psychedelic drugs and surmised that the psychedelic state might be characterized by an increase in integration compared to a normal waking state. Gallimore referenced a small study which employed quantitative electroencephalography to measure changes in gamma oscillatory power and coherence following ayahuasca ingestion and reported a highly integrated brain state (Stuckey et al., 2005). This finding is in accordance with an earlier study showing an increase of the gamma band power in ayahuasca users (Don et al., 1998). However, contrary to these results, other studies found generalized decreases in power across all frequency bands (Riba et al., 2002, 2004). While the neuropsychological correlates and clinical relevance of these electroencephalogram patterns remain to be elucidated, Gallimore’s work underscores the necessity of a comprehensive approach to ayahuasca’s pharmacology and the mechanisms of its cognitive, affective, and emotional effects.

The central nervous system effects of ayahuasca are distinct from our normal resting mental states sustained by the Default Mode Network as defined by specific brain areas which are activated when the person is at rest and/or not engaged in specific mental tasks. This Default Mode Network is usually active in meta-cognition, day-dreaming, reflecting on memories, but is apparently disabled by psychedelics (Carhart-Harris et al., 2014). Psychedelics alter this relaxed brain function by reducing cerebral blood flow and the oscillatory power in brain areas of the Default Mode Network that are typically synchronized and functionally connected. Ayahuasca decreases the functional connectivity within the prefrontal cortex and in connections with other areas of the brain that are involved in a wide range of ordinary cognitive processes (Palhano-Fontes et al., 2015).

This decoupling phenomenon results in increased flexibility of high-level networks involving a more open communication among them. It permits a freer operation of the medial temporal lobe structures, which are associated with the release of cognitive states closely related to emotions and fears. The outcome is a complex mental condition characterized by increased somatic awareness and subjective feelings, but lacking the metacognitive ability for self-reflection on personal behavior and one’s mentalization provided by the frontal cortex. This so-called primary cognition produces a state of heightened suggestibility because of the suspension of the frontal networks that are typically used to maintain control over mental processes and perceptions of the outside environment.

Bouso and Riba (2014) suggested that these varied effects of ayahuasca may also result from its ability to increase activity in various areas of the right hemisphere (anterior insula, anterior cingulate/fronto-medial cortex). These areas are implicated in somatic awareness, emotional arousal, feelings, and processing of emotional information. Ayahuasca also appears to increase activity in the left hemisphere’s amygdala/parahippocampal gyrus structures that play a role in emotional arousal and memory, enabling ayahuasca to make repressed memories conscious and to re-experience emotions associated with them. Such apperception enables one to reprocess these memories in more constructive ways and with a potential for processing traumatic pasts in novel ways.

## Neurochemical and Psychophysiological Mechanisms Proposed as a Basis for Ayahuasca’s Effects on Addiction

There are a variety of biochemical and physiological mechanisms through which ayahuasca can effectively address addictions (Prickett and Liester, 2014). The inclusion of two plant species in ayahuasca provides a variety of mechanisms for direct and indirect actions on both dopaminergic and serotonergic systems. Since the effects of DMT appear to reflect the general effects of tryptamines (e.g., DMT, LSD, bufotenin, psilocin, and psilocybin) some of therapeutic mechanisms would also be shared with these related substances. The effects of the harmine alkaloids, however, would be unique to ayahuasca. Separate studies with each of these chemical classes will be necessary to distinguish their different contributions.

Liester and Prickett (2012) proposed that “ayahuasca exerts anti-addictive properties via its direct and indirect actions on dopaminergic and serotonergic neurons in the mesolimbic pathway. Ayahuasca raises global 5-HT levels attenuating withdrawal effects and mitigating against potential dopaminergic excess when utilizing dopamine (DA) agonists. Ayahuasca balances DA in the MDP between the low levels associated with withdrawal and the elevated levels associated with initiation and reinforcement of addictive behavior” (Prickett and Liester, 2014). Therefore, the resolution of addiction through ayahuasca’s therapeutic potentials may act on four levels: (1) reducing brain DA level in the MDP through effects on the 5-HT receptors, which in turn (2) interferes with the synaptic plasticity. This neurochemical mechanism is supported by (3) psychological insights and processing of repressed traumas, enhancing decision making capabilities, which allowing the person to (4) examine first person transcendental experiences.

Additional neurophysiologic mechanisms for ayahuasca’s therapeutic effects involve neuroplasticity, the ability of neurons to alter their synaptic connections. Constituents of ayahuasca may affect brain derived neurotropic factor release through effects on the GABAergic and glutamatergic systems. These are involved in producing neuroplastic changes through triggering changes in gene expression which affect the architecture and communication between neurons. These exert effects on the existing neural circuits which mediate maladaptive addictive habits in stimulating the production of new circuits supporting new behaviors, with ayahuasca facilitating a neurological rewiring of the brain’s reward pathways. This model is supported by animal experiments (Oliveira-Lima et al., 2015) which demonstrated that ayahuasca not only inhibits early behaviors associated with the initiation and development of alcohol addiction, but also has long-term effects in preventing the reinstatement of ethanol-induced behavioral sensitization.

## Psychological and Psychosocial Effects of Ayahuasca

After an approximately 35 to 40-min latency period, the consumption of ayahuasca induces an intense modified state of consciousness that lasts approximately 4 h (McKenna, 2004). Perception, spatiotemporal orientation, beliefs about reality and the self, cognitive and emotional processes can all alter significantly during the experience. Visions of beautiful visual scenery are commonly reported together with some typical elements of the “ayahuasca world”: ayahuasca beings, power animals, spirit guides, tropical motifs, vibrant, and varying geometric patterns known from the literature of the cultural anthropology of shamanism. The neurological background of the strong visual effects has been revealed by functional magnetic resonance imaging studies as it was discussed above (de Araujo et al., 2012).

Considering the psychological therapeutic benefits, the emphasis is on the induction of an introspective dream-like experience characterized by visions, autobiographic and emotional memories which increase mindfulness capacities (Soler et al., 2015), as well as on the intellectual and spiritual insights gained during the encounters with ayahuasca. Ayahuasca experiences are a constant flow of mental contents, during which knowledge is gained by intuition rather than logic. They also show a high level of overall coherence. The level of self-reflection, reminiscence, ethical sensation, prosocial behavior (Frecska, 2008), creative thinking (Frecska et al., 2012), and redemption (de Rios et al., 2002) can be increased or elicited during the experiences. Various psychological blockages and denials may enter awareness and become illuminated from multiple perspectives allowing the participants to gain insight into their maladaptive behavioral, emotional and/or cognitive patterns. These psychodynamic contents are often accompanied by an enhanced internal moral attitude that forces the participants to face their deep thoughts and emotions that confront them with earlier wrongdoings, self-deceptions, and lies (Frecska, 2011). Repressed memories can surface causing emotional catharsis and opening the way to abreaction, relief, and remission. The strong serotonergic effect of ayahuasca and the internal phenomenon of being held or guided by an intelligent power can be considered responsible for the observation found in many personal reports that indicate that the retraumatization by the experience is avoided by reaching a certain flip-point, a “slew-round” to a previously inaccessible, corrective positive aspect of the emotional pattern. In such cases post traumatic growth becomes possible.

The emotional charge of ayahuasca experiences often follows a sine wave. Kjellgren et al. (2009) described an initial “contractile frightening state” during which participants frequently confront their innermost fears: fear of insanity, fear of death, paranoid thoughts or the despair of cosmic loneliness and outcast. Distressful somatic symptoms such as dizziness, diarrhea, nausea, and vomiting may also arise and become essential part of the process, carrying the subjective experience of purge and relief. If the subject is able to surrender, this unpleasant initial phase is usually followed by a sudden transformation of the experience into transcendental experiences, reflections, changed worldview and/or new orientation to life.

It is frequently reported—especially in what Metzner (Metzner, 1998) called hybrid shamanic and psychotherapeutic ritual setting—that participants arrive at ayahuasca ceremonies with a predefined personal intention such as asking for healing, guidance, teaching or a solution to a personal problem. These intentions seem to support the experience by two means. They seem to provide a basic structure to the unconscious materials that arise during the experience as well as a narrative frame for the interpretation and integration of the experience.

Scientific interest toward ayahuasca has grown rapidly over the last decades and so the most pregnant directions of its possible therapeutic use have begun to be outlined. However, the illicit legal status of the brew that ensues from its DMT content imposes heavy impediments to its scientific understanding. Many findings therefore come from investigations carried out among the members of the Brazilian syncretic churches, where the legal use of ayahuasca primarily serves religious aims instead of therapeutic ones.

The Hoasca Project was first to provide scientific findings regarding the psychopharmacological and psychological effects of ayahuasca (Grob et al., 1996). The study involved 15 members of the *União do Vegetal* and suggested that the long-term use of ayahuasca resulted in positive behavioral and lifestyle changes in the participants’ lives. An unexpected finding was the possible anti-addictive effect of the brew as revealed from the reports of several participants of the study who had struggled with alcohol and substances abuse before having joined the *União do Vegetal*. Two decades later a similar study involving 32 members of *Santo Daime* arrived at the same conclusions (Halpern et al., 2008).

Barbosa’s team (Barbosa et al., 2005) concluded that the use of ayahuasca increased assertiveness, joy of life and liveliness among the members of the Brazilian syncretic churches, while da Silveira and others (da Silveira et al., 2005) found that adolescents who regularly consume ayahuasca show less signs of anxiety, are more optimistic, self-confident, insistent, and emotionally mature than their peers. Similarly, results of psychometric tests applied by dos Santos’ group (dos Santos et al., 2007) revealed that after ayahuasca use, individuals respond with less anxiety to states that involve hopelessness and resemble panic, while measures of state- or trait-anxiety remain unaffected. Recently Barbosa’s team (Barbosa et al., 2012) performed a meta-analysis on publications listed on PubMed with the aim to summarize current knowledge about the effect of ayahuasca on health. It concludes that the consumption of ayahuasca is safe and under certain conditions may even be beneficial. Results of a longitudinal prospective study on a large population of regular users showed no signs of cognitive impairment and the decoction had no negative effect on coping strategies or the general mental health (Bouso et al., 2012). While there are occasional reports of ayahuasca users dying during the episode, they typically reflect underlying health conditions or prolonged neglect of participants during rituals.

The prolonged social contact among participants that is typical of ayahuasca based treatments provides the opportunity for developing the social support that is crucial to recovery from many mental illnesses including addiction. The ceremonial context enhances bonding among participants that can facilitate therapeutic processes, especially through the provision of social support and the enforcement of social norms that encourage an abstinent lifestyle. The participants in ayahuasca ceremonies of the churches provides social support for managing stress and gives a sense of belonging that motivates lasting behavioral changes.

Since DMT is known to be a very potent 5-HT agonist, it can decrease impulsive behavior and facilitate social interactions (Frecska, 2008). A rapid 5-HT receptor action can explain the traditional indication of ayahuasca use in crisis prevention and occasioning redemption (de Rios et al., 2002). The prosocial, cohesive action effect of ayahuasca is reflected in the quality of the elicited subjective experience, which commonly involves ethical lessons (Shanon, 2002). Ayahuasca is highly revered by mestizo curanderos as a stern moral teacher (Luna, 1986).

## Psychotherapeutic and Spiritual Effects of Ayahuasca

Ayahuasca experiences often reflect psychodynamic effects that contribute important therapeutic outcomes through providing a connection with significant aspects of the person’s past, elevating repressed memories into consciousness where they can play a role in psychological healing through restructuring. A frequent theme mentioned by victims of abuse and recovered addicts is that the ayahuasca-induced visions helped them to recover long-forgotten memories of traumatic events that they were then able to work through, providing a basis for restructuring their personal life (Loizaga-Velder and Verres, 2014). Ayahuasca-induced insights facilitate self-reflection, producing changes in self perspectives that can trigger psychodynamics insights which provide solutions to personal problems that underlie maladaptive lifestyles. Ayahuasca helps resolve personal conflicts by providing conscious insights into patterns of psychological functioning that underlie pathological behaviors such as substance abuse and dependence. Participants of ayahuasca rituals often report insights that enable acceptance of previously denied problems and dysfunctional patterns. The visionary state of consciousness produced by ayahuasca can also provoke reflections on personal relationships which provided the motivation for making the changes necessary to resolve interpersonal problems.

Hence, ayahuasca’s effects appear to evoke psychodynamic mechanisms and psycholytic effects that can augment access to pre-conscious and unconscious memories. This release of repressed emotions can catalyze healing processes by contributing to the resolution of traumas by releasing the person from dysfunctional habits that underlie the dynamics of addiction and many other behavioral problems. Psycholytic processes engendered by ayahuasca also promote an awareness of the likely future outcomes and personal consequences of maladaptive behaviors, providing a motivation for change. Personal accounts of addicts reveal that the ayahuasca experiences led many of them to perceive that their drug use was leading them down a path of self-destruction that would lead to their death. Ayahuasca might produce death experiences, sometime a sense that one was dying, or a vision of oneself as dead as a consequence of drug use. These experiences led to realizations that helped them to make radical changes in their behavior by providing additional motivation to make necessary changes in personal behavior and lifestyle (Loizaga-Velder and Verres, 2014). A basic effect of ayahuasca on psychological process involves a confrontation with oneself, forcing a greater personal awareness that facilitates a reconstruction or restructuring of the nature of oneself (Fernández and Fábregas, 2014). A reassessment of the past provides the basis for an experience of cleansing from the past events and the basis for new perspectives into one’s patterns of behavior.

Speculating on the psychotherapeutic effects of ayahuasca, Naranjo (1979) suggested that its effects are similar to that of an intense psychotherapy. He attributed the prime therapeutic effect of ayahuasca to its harmaline content instead of DMT. Trichter (2010) claimed that the therapeutic effect reported in personal anecdotes results from the psychological work being carried out at a much deeper level than in the case of traditional psychotherapeutic methods. Mabit (2007) listed eleven factors that contribute to the brew’s therapeutic effect, one of which is its ability to lower defenses and to reveal ego defense mechanisms. This in turn allows repressed unconscious materials to enter consciousness and extinguish the emotional charge of past traumatic experiences, helping the participants to temporarily experience thus far unknown emotional states and cognitive patterns; and through them they better understand the means and directions of adjustments needed in their lives.

Maté (2014) proposed that ayahuasca is capable of treating many conditions because both physical and psychological conditions can be based in unconscious psychological conditions. Psychedelics assist by bringing these dynamics into consciousness, initiating a process of liberating the person from these influences. Maté (2014) also suggested that while deep psychological dynamics may emerge into awareness during ayahuasca ceremonies, their therapeutic potential depends on trained guidance to bring these potentials to fruition. Successful treatment with ayahuasca requires an experienced person to provide structure and guidance to effectively orient to the visions, the therapeutic purpose, and the development of the experience across sessions. Lacking qualified assistance in achieving their full integration, important experiences may not produce benefits. Nonetheless, he emphasizes that in the right supportive circumstances, ayahuasca can help provide the insights and personal meanings that can help resolve the underlying dynamics of addiction by triggering visions of the emotional states and traumatic imprints.

Frecska (2011) supposes that the experience consists of repeated sequences of deconditioning and reintegration. This can be conceived as a “secure” form of regression, which makes the correction of maladaptive cognitive and emotional structures (personal network of concepts, maladaptive patterns, etc.) possible. Echenhofer (2012) endeavored to draw parallels with experiences from other spiritual traditions and divides the process of deconditioning and reintegration into three subphases: (1) form dismantling and healing, (2) form creation, and (3) form expression.

Ayahuasca also produces transcendent and mystical experiences, the “peak experiences” that led to the “psychedelic” paradigm of LSD treatment that was based in recognition that these substances provide an effective treatment for alcoholism by changing the individual’s personal awareness, self-perceptions and worldview. A significant dimension of the spiritual experience was a transformation of personal consciousness in ways that eliminated the craving for drugs. Mystical or spiritual experiences reported during the ayahuasca sessions are frequently said to have a life changing effect on those bearing them, sometimes setting them off on a path of spiritual mission (Krippner and Sulla, 2000).^3^ Although subjective accounts have limitations as they are vulnerable to memory distortion and self-defense mechanisms, the high rate of such reports is remarkable. Furthermore, these early observations are in line with the experimental findings of Griffiths’ team at the Johns Hopkins University (Griffiths et al., 2011) using psilocybin. By analyzing the reports of many 100s of ayahuasca experiences Shanon (Shanon, 2002) came to the conclusion that the experiences can sometimes have such a deep effect that the individuals may feel they are no longer the same person.

The psychotherapeutic effect, however, does not only depend on the experience and its phenomenal content or depth. In addition, it depends on how much the insights gained during the experiences become integrated into the everyday life of the participants afterward. Without adequate integration any experience loses its therapeutic potential in time. House (2007) warned that psychedelic experiences can carry the feeling that the desired psychological change happened during the experience itself. Such feelings are, however, illusionary and by diverting the participants from real integration they may cause more harm than benefit.

Another possible pitfall emphasized by Trichter (2010) is the phenomenon of spiritual bypassing, which occurs when individuals escape into repeated spiritual practice in order to avoid their psychological blockages. As a result an unhealthy relationship may be developed with the given spiritual tradition and techniques, which disguises the real psychological problems. The integration of the experiences can best be supported by psychotherapy carried out by an expert. Given the uncertain legal status of ayahuasca in many countries, however, this is only possible afterward, separate from the ritual (Jungaberle et al., 2008; Oak et al., 2015). Such professional assistance for the integration is, however, not at all straight forward since the number of therapists who are familiar with handling such unusual experiences is generally very low. Considering that the number of people seeking out ayahuasca ceremonies keeps growing every year, the rate of related scientific research and professional education has become crucial and requires attention.

## Use of Ayahuasca in the Treatment of Addictions

The potentials of ayahuasca administration in substance use disorders illustrate the necessity of an integrative view spanning from the biological to spiritual levels. In recent years there has been a growing scientific interest in the possible therapeutic use of ayahuasca in the treatment of addictions. Various forms of clinical research have attempted to reveal the mechanisms that lie under the commonly reported anti-addictive effects of ayahuasca (see Winkelman, 2014 for review). Loizaga-Velder and Pazzi (2014) revealed the bases of ayahuasca therapies with an empirical study of therapists who used ayahuasca professionally in addictions treatment and addicts who participated in ayahuasca treatment programs based on indigenous shamanistic approaches. These authors noted that most patients reported therapeutic effects involving: “augmenting body awareness, reducing drug-craving, triggering different types of emotional processes (catharsis, perception of previously suppressed emotions, generating inner resources for coping with emotions or urges to use), supporting introspection (self-analysis, eliciting awareness of addiction, and its adverse effects on oneself and others), and enhancing self-efficacy (becoming aware of positive aspects of oneself, thus improving self-esteem and confidence to stay sober)” (Loizaga-Velder and Pazzi, 2014).

Successful treatment of addictions involves a range of physical effects of ayahuasca, including the purgative and emetic effects, bodily sensations, the evoked visions and the recall of significant past memories. The physical experiences produced are interpreted within the ritual context, one’s personal circumstances and with respect to the recovery processes. Ayahuasca activates a body-oriented dynamics that provides relief from stress, as well as a detoxification experience derived from the emetic effects which may produce an attenuation of craving. Within the shamanistic traditions, the vomiting or purge is seen as a part of the process of detoxification of the body, not only the elimination of toxins, but also the expulsion of morbid emotional and mental conditions. Loizaga-Velder and Pazzi (2014) noted that many report that the emetic effect experienced through vomiting is seen as providing an elimination of troublesome emotions as well as provoking diverse emotional dynamics that trigger psychological and spiritual reactions.

One important institution where ayahuasca is applied therapeutically is the Takiwasi Center for Rehabilitation of Drug Addicts and for Research on Traditional Medicines in Tarapoto, Peru (Mabit, 2007). The program integrates traditional ayahuasca rituals with physical, psychological, and spiritual activities into treatments that address a range of factors contributory to addiction. Founded in 1992 by Jaques Mabit, a French medical doctor, the Takiwasi uses a mixed approach of Western psychotherapy and traditional Amazonian medicine based on local herbs. The success is attributed not to the ayahuasca alone, but the use of other plants, the ritual setting, the community life and the interactions with therapists. Within the Takiwasi program the traditional medicines and rituals are combined with transpersonal psychology and modern social techniques to guide the personal transformation of addicts, using the ayahuasca ritual to produce profound alterations of mind that change addicts’ outlook on life, on their spiritual strength and faith.

The Institute of Applied Amazonian Ethnopsychology (see Fernández and Fábregas, 2014) is another group which has applied shamanistic perspectives to the treatment of addictions with ayahuasca. The approach is based on a minimalist model, without extensive focus on ritual healing processes, but nonetheless integrates influences from the Brazilian *Santo Daime* religion, shamanism, Eastern meditative disciplines and transpersonal psychology. The approach also includes other healing techniques outside of the ayahuasca sessions such as: “individual psychotherapy; workshops on emotions, breathing techniques, psychodrama, and bibliotherapy; and family constellation therapy [and] … other practices such as massage, colonic irrigation, shiatsu, and naturopathy” (Fernández and Fábregas, 2014). While the community context is used to counter the extreme self-centeredness of the addict, the treatment also involved solitary periods during ayahuasca sessions when the participants would retreat to their own cabins for periods of introspection, self-reflection and engaging their own healing processes based on self-regeneration from within the person.

Some centers have developed structured studies for monitoring their therapeutic outcome. In an observational study Thomas’ group (Thomas et al., 2013) followed 12 members of a Canadian First Nations community, a subpopulation especially prone to addictions due to a mixture of social and psychological factors including transgenerational historic traumas and cultural dislocation. These authors found that participation in ayahuasca retreats correlated with enhanced mindfulness, personal empowerment, hopefulness, improvements in quality of life, and increased subjective feelings of connection with self, others, spirit and nature as rated with the Difficulty in Emotion Regulation Scale, Hope Scale, Philadelphia Mindfulness Scale, and the McGill Quality of Life questionnaires. In addition, the four Week Substance Use Survey also suggested that this form of ayahuasca-assisted group therapy might be associated with reductions in substance use, particularly reductions in problematic cocaine use.

Cross-sectional and longitudinal case-control studies showed that ritual and religious ayahuasca users present fewer alcohol-related problems than control groups and that illicit drug use diminished after joining ayahuasca churches (Grob et al., 1996; Doering-Silveira et al., 2005; Halpern et al., 2008; Fábregas et al., 2010). It is an open question whether ayahuasca has anti-addictive properties *per se* or if the social factors (e.g., religious social reinforcement) are the primary factor in producing these results. However, known neurochemical and psychophysiological actions of ayahuasca constituents provide evidence that pharmacological factors are a significant feature of ayahuasca’s anti-addiction effects.

## Discussion

Evaluating the health benefits and risks of a remedy of plant origin is more difficult than that of assessing synthetized compound. In the case of ayahuasca such an evaluation is also complicated by the admixture nature of the brew, the strong psychoactive effects, the setting of its typical administration, and by the legal and financial impediments. As far as the plant components of the brew are concerned, significant differences in health potentials may stem not only from the particular species of the DMT plant being used (*Psychotria virid* is or *Diplopterys cabrerana*), but from the variety of the *B. caapi* (e.g., *cielo, trueno*, black, or red, etc.) in the mixture. A range of interactions may emerge between the plant alkaloids. Among those the most important occurs between the β-carbolines MAO inhibitor effect and the DMT content. MAO activity may vary considerably between individuals and across sessions (for stress-related and endogenous inhibitor mediated changes; see Obata, 2007). Since the enzyme may not be fully saturated by the competitive inhibitor, or it can be overloaded with other monoamines, the same DMT content may result in different plasma levels. Moreover, the same plasma DMT level can lead to different intraneuronal concentrations depending on the efficacy of the active DMT-uptake processes into the brain, neurons, and vesicles (reviewed in Frecska et al., 2013). The ayahuasca’s effect on suggestibility interferes with the conditions of set and setting (Barbosa et al., 2005) and favors stronger placebo response. To make this issue more difficult: ayahuasca cannot be camouflaged easily for double-blind administration. While in most of the countries ayahuasca is not scheduled as rigorously as its DMT constituent, this doesn’t mean that its legal status is unambiguous and it can be studied on par with other plant remedies (St. John’s wort, for example). Plant medicines do not enjoy the financial support of pharmaceutics introduced and promoted by the industry. Patenting ayahuasca would violate indigenous people’s right over their intellectual heritage and such use could constitute biopiracy; these circumstances further undercut potential financial interest and industrial support.

The wide spectrum of ayahuasca’s effects—as was outlined in the text—from biological to psychosocial (even spiritual) may also complicate the issue, but on the other hand it makes this plant remedy more interesting and promising for future explorations. The Sig-1R mediated action places ayahuasca research in the new stream of medical investigations, namely, it fits nicely into the emerging unifying concept of systemic and degenerative illnesses. It would be intriguing to see how a shamanic potion can be useful against wide array of civilization diseases. Ayahuasca-induced psychological effects like increased insight, reframing of cognitive structures, increased imaginary, and cathartic emotions promise potentials for ayahuasca use in psychedelic assisted psychotherapy by means of facilitating interventions based on insight oriented, cognitive, guided affective imagery, and cathartic techniques (Loizaga-Velder, 2014). The anti-impulsive, prosocial, cohesive action of ayahuasca should be tested in detention centers with young delinquents, and there is another important field of interest: combat-related post-traumatic stress.

Social isolation, lack of trust, violent outbursts, emotional numbing, and vivid recollection of traumatic experiences are often present in PTSD, a condition difficult to treat. Combat-related PTSD patients have been significantly less responsive to conventional therapies than other PTSD populations (e.g., victims of rape, survivors of natural disasters, participants of life-threatening accidents, or members of rescue-teams facing mass casualties). Veterans with PTSD may require treatment tailored to the unique nature of combat, military culture, and their individual circumstances (Yehuda, 2008). Generally, combat-related traumatic experiences can be complicated by aggravating factors: combat veterans are typically multi-traumatized over an extended period of extremely distressful time, frequently have survival guilt, guilt over killing enemy combatants, causing collateral damage, being responsible for friendly fire, and they usually witnessed the dismemberment and death of their comrades (Frecska, 2011). Our hypothesis is that—similar to methylenedioxy-methamphetamine (MDMA) which has already been tested (Mithoefer et al., 2013)—ayahuasca can also improve treatment of PTSD through enhancing trust and social feelings. In addition to these beneficial core effects, in proper settings it may also elicit “moral lessons” with subsequent relief and redemption. Additionally, the induced visions can be integrated into a cognitive re-exposure therapy with desensitization and reprocessing. The latter is the most effective cognitive treatment recognized by the National Center for PTSD. Ayahuasca facilitated cognitive exposure therapy could possibly cut down the long desensitization period necessary in the traditional psychotherapeutic approach.

The ideal scientific assessment of ayahuasca therapies are admittedly lacking, with the double-blind clinical studies a practical challenge. From the perspective of medical and research ethics, the lack of such studies should not be seen as reasons for absolutely prohibiting treatments with ayahuasca, however. In order to make our point, we would like to refer to the statements of Sackett et al. (1996), who warned for an intricate approach toward evidenced based medicine: “Evidence based medicine is the conscientious, explicit, and judicious use of current best evidence in making decisions about the care of individual patients. The practice of evidence-based medicine means integrating individual clinical expertise with the best available clinical evidence from systematic research” (Sackett et al., 1996).

In short, evidence-based medicine is the integration of experience collected in clinical practice with evidence gained from trial based research (evidenced based medicine = trial based medicine + clinical practice). Indeed, Sackett and his coworkers (Sackett et al., 1996) go on: “Evidence based medicine is not restricted to randomized trials and meta-analyses. It involves tracking down the best external evidence with which to answer our clinical questions.” and “…some questions about therapy do not require randomized trials (successful interventions for otherwise fatal conditions) or cannot wait for the trials to be conducted. And if no randomized trial has been carried out for our patient’s predicament, we must follow the trail to the next best external evidence and work from there.” Not only the ayahuasca treatment programs for drug addicts have shown considerable expertise on the side of clinical practice, but formal assessments cited above support therapeutic ayahuasca use under certain conditions. Therefore, while randomized controlled trials are needed, these should not be seen as an absolute necessity to justify further use. In our opinion, the studies that are available do justify the application of ayahuasca at least in the treatment of addictions.

Randomized clinical trials typically use double blind controls to assess the role of placebo or expectancy effects. The use of double blind controls is particularly problematic for ayahuasca. Ideal double-blind clinical trials may be forever beyond the methodological possibility given the ritual elements. Double-blind clinical studies will be particularly challenging in terms of appropriate pharmacological and ritual control conditions. What would constitute a placebo substitute for ayahuasca might be found, but the ritual aspect that is implicated in treatment success also requires some control. But such studies are to separate drug effects from set and setting—such as ritual. It is, however, these combined physiological and ritual elements of ayahuasca that appear to have the effects on treatment outcomes. The shamanistic approaches attribute therapeutic effects to a variety of factors, including pharmacological as well as psychological, but most significantly the interactions of the biological and personal levels with the spiritual. For example, the evidence for ayahuasca as an effective treatment for addictions is from practices that espouse a view that emphasizes the interaction of a sacrament with physiological effects with the interpersonal dynamics of ritual. Consequently, the more one agrees to the importance of addressing the spiritual aspects of human experiences as part of therapy by extending the Engelian paradigm (Engel, 1977) to the biopsychosociospiritual model (Bishop, 2009), the more one should adhere to the clinical practice side of the Sackett’s equation, since targeting spiritual levels is beyond current scientific approaches.

Nevertheless, studies on ayahuasca as a therapy can attempt to isolate the physiological effects from ritual by controls. Double blind studies will also be necessary to determine the effects of the separate components (DMT vs. harmine) as well as their interactions. These should have to incorporate additional controlled trials to determine long term risks and benefits, and relative effectiveness in comparisons with other procedures or agents used to treat addictions.

To date we lack controlled ayahuasca and DMT studies largely due to administrative prohibitions. The necessary phases of evaluation for the psychedelic medicines have to rely on an integration of various forms of validation through a “triangulation” that combines data derived from: animal research; epidemiological research on general risks and adverse reactions; medical case studies and personal accounts of those who have received these substances as treatments; and process oriented research that assess pre and post-treatment conditions with a variety of standardized tools—as Alper and Lotsof (2007) pointed out in their assessment of ibogaine. While administrative regulations considerably impede research, they have not entirely obstructed it, but in terms of clinical applications they have been absolutely prohibitive. We would argue, however, that although treatment with these substances is ruled out by their Schedule I classification, their use as remedies is justified in that they reduce the patient’s severe suffering without causing disproportionate harm to others (i.e., other people, or to the State’s interest).

We are not going to see major pharmaceutical companies address the need for evaluation of ayahuasca or other plants as medicines. There are no financial incentives. So how can physicians and patients make use of such treatments? In addition to patients’ predicaments their choices and preferences are other factors which should not totally be ignored in good clinical care. There are multiple levels of society at which we need to act to change the current political climate that regulates these substances. Public pressure on federal regulatory agencies is central to advancing experimental and treatment use of these substances. This influence involves general education, education of the media, and activities in public health and policy organizations, as well as private funding of research and corporate developments. The arguments from the cumulative scientific, clinical, ethnographic and cross-cultural evidence regarding the immense potentials of these substances are the basis for a public education approach to facilitate professional development, media coverage and popular pressure upon the government to change federal regulations and procedures. In summary, education, public policy development, and collective political action, rather than just more science, is necessary for changing opportunities for the use of ayahuasca in treatment of some of the most ravaging social diseases of our times.

## Author Contributions

All authors made significant contributions to the preparation of the manuscript and approved it before submission. EF developed the paper design, wrote the biological section, edited the References, and finalized the manuscript. PB wrote the first draft of the manuscript and elaborated on the psychological components. MW worked on the addictological aspects and revised the style of the manuscript.

## Conflict of Interest Statement

The authors declare that the research was conducted in the absence of any commercial or financial relationships that could be construed as a potential conflict of interest.

## Footnotes

1 The purpose of traditional ayahuasca use has been to heal both physical and spiritual ailments. *Mestizo curanderos* consider ayahuasca as a plant teacher Luna (1984). DMT has been scheduled worldwide as an illicit drug with no medical use. Due to their DMT and/or β-carboline content ayahuasca constituent plants are included as controlled substances in several countries (i.e., Australia, Bulgaria, Canada, France, Lithuania, Poland, Switzerland, and Ukraine). Ayahuasca’s classification in France as *stupéfiant* illustrates well the discrepancy with *Mestizo* views.

2 Based on more and more converging evidence, it is tempting to speculate that the “Grand Unification Theory” (GUT) of medicine can be based on the gut. According to this unifying concept, a “leaky gut” lets some gut contents (e.g., bacterial endotoxins) to enter circulation and shifts the anti-inflammatory ↔ pro-inflammatory cytokine balance to pro-inflammatory direction. A long-standing pro-inflammatory condition with the simultaneous oxidative, mitochondrion, and ER stress (see in the text) can change the permeability of other membranes in the body (e.g., blood-brain barrier, synovial stratum, endothel, bronchial mucosa, and/or alveolar wall). This way the permeability problem of the gut extends to other barriers, and a universal membrane deficiency may serve as a common ground of many illnesses of civilization.

3 This was exactly the way every Brazilian syncretic church *União do Vegetal, Santo Daime*, and *Barquina* got started!

## Abbreviations

DMT: dimethyltryptamine
ER: endoplasmic reticulum
LGI: low-grade inflammation
MAM: mitochondrion-associated membrane
MDP: mesolimbic dopamine pathways
PTSD: post-traumatic stress disorder
Sig-1R: sigma-1 receptor
UPR: unfolded-protein response
